# Prevalence and associated factors of worry for cancer in patients with a Barrett’s esophagus

**DOI:** 10.1038/s41598-024-53428-y

**Published:** 2024-02-04

**Authors:** M. C. M. van der Ende-van Loon, R. A. B. Oude Nijhuis, W. L. Curvers, E. J. Schoon

**Affiliations:** 1 Department of Gastroenterology and Hepatology, Catharina Hospital, Michelangelolaan 2, 5623 EJ Eindhoven, The Netherlands; 2 Department of Gastroenterology and Hepatology, Amsterdam UMC, Amsterdam, The Netherlands; 3 GROW: School for Oncology and Developmental Biology, Maastricht UMC, Maastricht, The Netherlands

**Keywords:** Cancer, Gastroenterology, Digestive signs and symptoms

## Abstract

Although the risk of cancer progression in a Barrett’s esophagus (BE) is very low, worrying about cancer is known as an important factor affecting HRQoL. The aim of this study was to determine the proportion of BE patients with high levels of worry for cancer, to compare outcomes of patients endoscopically treated for BE neoplasia (DBE), non-dysplastic BE patients (NDBE) and patients with reflux symptoms, and to examine associated factors. We performed a cross sectional, exploratory, self-administered questionnaire study using the cancer worry scale, and the reflux disease questionnaire. A total of 192 DBE patients, 213 NDBE patients and 111 refractory reflux symptom patients were included from October 2019 until July 2021, 76.8% of BE participants were male and aged 66.9 years. High cancer worry was reported in 40.6% of the DBE patients and 36.2% of NDBE patient. Reflux patients scored statistically significant worse with 56.6% stated high cancer worry. Positive correlations were found between reflux symptoms and cancer worry in NDBE patients and reflux patients. In DBE patients’ negative correlations were found between higher cancer worry and younger age as well as a family history of esophageal carcinoma. A clinically significant group of BE patients reported high cancer worry, which was associated with reflux symptoms in NDBE patients and a younger age and a (family) history of esophageal carcinoma diagnosis in BE patients treated for (early) neoplasia. Physicians should communicate about the actual cancer risk, which leads to greater patient understanding and therefore may have a positive impact on health outcomes.

## Introduction

Cancer is among the leading causes of death worldwide. In 2018, there were 18.1 million new cases and 9.5 million cancer-related deaths worldwide^1^. Cancer has been one of the most feared diseases for years^2^. Contrary to the negative image among the general public, epidemiological analyses show that cancer survival rates are gradually increasing. Comparable with numbers in Europe and the United States of America, the 5-year survival rate for esophageal adenocarcinoma (EAC) in the Netherland has risen from 8% in the early 1970s to 23% currently. In the past decades, substantial progress has been made in the diagnoses and treatment of EAC. The best chance for improved survival of patients with EAC remains detection of the cancer at an early and possibly curable stage. The main cause from which EAC can develop is the premalignant condition Barrett esophagus (BE). BE is a complication which occurs in about 10% to 15% of people with chronic or longstanding gastroesophageal reflux disease. The diagnosis of BE is made if the distal esophagus is lined with columnar epithelium with a minimum length of 1 cm (tongues or circular) containing intestinal metaplasia at histopathological examination^3^. While pre-malignant conditions that are not under surveillance may eventually become cancer, in many cases the chances of progression is very low. Among patients with a BE, approximately 5% will develop EAC ultimately^4^. Previous studies have shown that it is difficult for individual BE patients to accurately estimate their cancer risk^5–7^. Therefore, a diagnosis such as BE, may cause anxiety and worry.

Over the past ten years, non-invasive endoscopic treatment (ET) techniques such as endoscopic resection (ER) or radio frequency ablation (RFA) have become the preferred treatment strategy for the removal of early neoplastic lesions (high grade dysplasia (HGD) and early EAC). Although, ET have shown to be effective for eradication of BE related neoplasia with remarkably low recurrence rates of neoplasia^8^, high numbers of worry for cancer are descripted in the literature. Studies have shown that worry for cancer in patients before, and within 12 months after ET is high and comparable to those who have never had dysplasia^9–11^. However, little is known about the factors that influence these worries about cancer. For example, it is not clear whether actual risks for developing EAC (such as BE length and histology) actually increase cancer worry. In fact, a long-term follow up study^12^ found endoscopically treated patients had statistical significantly higher levels of worry for cancer and general anxiety than surgically treated patients**.**

A previous systematic review identifying the key factors associated with fear of recurrence among cancer patients found there was strong evidence for an association between physical symptoms and fear of cancer recurrence^13^. Although previous studies found the majority of the BE patients reported good reflux symptom control^7,14^, reflux symptoms are known as an important factor for negative illness perception on BE^14^. In addition, it appears that patients who overestimate their cancer risk tend to experience more symptoms of reflux^7^. Due to the small number of studies on worry for cancer in BE patients, knowledge on factors associated with worry for cancer in BE patients is lacking. Identification of associated factors could help physicians to identify BE patients at risk of experiencing high levels of cancer worry. In order to better understand the impact of ET on cancer worry, it is important to investigate the level of cancer worry in a group of BE patients endoscopically treated for (early) neoplasia and in patients without neoplasia who are included in an endoscopic surveillance program. In addition, it is important to explore the potential impact of the label of Barrett's diagnosis and the presence of physical symptoms.

The aim of this study was to determine the proportion of BE patients with high levels of worry for cancer and to compare outcomes of patients endoscopically treated for BE neoplasia (DBE) and non-dysplastic BE patients (NDBE) with a non- BE control group of patients with reflux symptoms, and associated factors are studied. We hypothesized that the minority of BE patients would experience high cancer worry which would be associated with physical symptoms and not related to factors that would actually increase the risk of cancer such as Barrett’s length or histology outcomes.

## Method

This was a cross sectional, exploratory, self-administered questionnaire study assessing worry for cancer in patients with a BE and refractory reflux symptoms. Patients were included from a single, tertiary referral centre for surveillance and endoscopic treatment of BE, the Catharina Hospital Eindhoven, the Netherlands. Participants completed the questionnaire before their endoscopy appointment from April 2018 until March 2022. Due to the COVID-19 pandemic, inclusion was interrupted between January 2021 and July 2021.

### Dysplastic Barrett’s esophagus (DBE) group

This first group of patients had a history or presence of confirmed low grade dysplasia, high grade dysplasia or EAC (defined as R0 endoscopic resection of a pT1a or pT1b adenocarcinoma) in histology prevalent BE and treated with at least one endoscopic procedure, e.g. endoscopic submucosal dissection (ESD), endoscopic mucosal resection (EMR) or radio frequency ablation (RFA). Patients were excluded when treated with a surgical esophageal resection, R1 endoscopic resection, and patients who underwent neoadjuvant/adjuvant chemotherapy or radiation as part of treatment of EAC.

### Non Dysplastic Barrett’s esophagus (NDBE) group

The patients in this second group were recruited from an endoscopic surveillance program for BE. All patients had proven macroscopic (metaplastic columnar epithelium above the gastro-esophageal (≥ 1 cm) junction, which was clearly visible endoscopically) and histologic (presence of intestinal metaplasia confirmed from esophageal biopsy) NDBE. Patients were excluded if there was presence of low-or high-grade dysplasia or EAC in BE histology.

### Refractory reflux group

The group contained of patients with reflux symptoms referred for an upper endoscopy. In these patients symptoms of heartburn, regurgitation, and/or chest pain were present for at least three months and three times a week^15^. Patients used a standard-dose of Proton-Pump inhibitors (PPI) therapy for at least three months with a minimum of three times a week. Patients with pre-existing esophageal disorders or BE were excluded.

At the time of completing the questionnaire, all participants were above 18 years of age. Furthermore, patients were able to read, understand and complete the Dutch informed consent form and the study questionnaires. Patients were invited to participate with a postal invitation and received a one-time postal reminder when they did not respond after four weeks.

### Questionnaires

The questionnaire asked participants to complete baseline items on age, gender, employment status, educational level, and comorbidity (diabetes, arthritis, mental illnesses, cancer, and diseases of hart, neurology, kidney, lung, and skin). In addition, data on the previous performed ET (date of procedure, histology and length of BE) were obtained from the medical record of the DBE patients.

Worry for cancer was assessed using the Cancer Worry Scale (CWS). The CWS is used in research to assess concerns about developing cancer or cancer recurrence and the impact of these concerns on daily functioning^16^. The CWS was translated in Dutch by Douma and colleagues^17^. The six items of the CWS are rated on a 4-point Likert scale ranging from “never” to “almost always”. Scores range from 6 to 32, with a higher score indicating more fear of cancer. Based on a previous Dutch validation study, patients were divided into three categories: no cancer worry (score < 6), low level of cancer worry (score 7–9), and high level of cancer worry (score ≥ 10)^16^.

The presence of reflux symptoms was measured using the Reflux Disease Questionnaire (RDQ). Extensive research has found this questionnaire to be reliable, valid, responsive and above all practical^18^. Furthermore, the RDQ outcome seems to correlate well with quality of life^19^. A Dutch validation study showed the RDQ is a valid and reliable questionnaire with excellent construct validity and a good relationship to quality of life^20^. RDQ includes 12 items assessing the frequency and severity of heartburn, acid regurgitation and dyspeptic complaints, which are scored on a 5-point Likert scale. The mean of all three dimensions gives a total score ranging from 0 to 5. Where a score of 0 represent nil symptoms, a score of 1–2 mild symptoms, and 3–5 severe symptoms of reflux^21^.

### Analyses

Continuous sociodemographic data, are presented with means and standard deviation (SD). Categorical variables are summarized with frequency and percentages(%). The DBE patients were allocated according to the time from the last ET (respectively 0–5, 6–11, 12–35, and > 36 months), the worst pathology found (LGD, HGD, EAC and high risk EAC). A high risk EAC was defined as EAC with at least SM1 invasion or vascular invasion. NDBE patients were distributed according the length of their BE (< 10 cm and > 10 cm).

To answer the first research question, which was: what is the proportion of BE patients with high levels of worry for cancer? The scores of the CWS were divided into three categories: no cancer worry (score < 6), low level of cancer worry (score 7–9), and high level of cancer worry (score ≥ 10).

The second research question was to investigate what the differences are on cancer worry and reflux symptoms between patients endoscopically treated for BE neoplasia (DBE), non-dysplastic BE patients (NDBE) and a non- BE control group of patients with reflux symptoms. Therefore, a one-way ANOVA was first was used to determine differences between the three patient groups (DBE, NDBE, refractory reflux). Then a post-hoc test was performed to identify differences on outcomes between the BE groups DBE and NDBE. Finally, a student t-test or Mann Whitney U (depending on normality), and the Chi-Square test for categorical variables were used to identify differences between all BE patients (DBE and NDBE) and the reflux control group.

For the final research question on exploring which factors were associated with worry for cancer, Spearman's rho or Pearson 'r (depending on continuous or categorical variables) were used. The outcome variable was total CWS score and the dependent variables: gender, age, marital status, employment status, total comorbidities, positive history of cancer, positive family history with cancer, months after ET, worst pathology and BE duration. Statistical analyses were performed using IBM Statistical Package for Social Sciences (SPSS) software (version 25). In this explorative study, significance levels were set at the 0.05 level (two-sided).

### Ethics approval and consent to participate

All subjects gave written informed consent in accordance with the Declaration of Helsinki. The protocol was approved by the Medical Ethical Committee United (MEC-U) with reference W19.068.

## Results

The questionnaire was completed by a total of 405 BE patients: 192 DBE patients (response rate 60.1%) and 213 NDBE patients (response rate 60.3%). Sociodemographic characteristics of all patients are presented in Table 1. The mean age of all BE patients was 67.1 years and the majority (77%) of participants were male. There were statistically significant more men included in the DBE group in comparison to the NDBE group (X^2^(2) = 11.78, p.001). There were no other differences between the two BE groups on sociodemographic characteristics. Just under half of the DBE patients previously treated with ET, had a follow-up of more than three years. No differences were found in terms of baseline characteristics within the DBE groups when allocated on histology and time from ET.

**Table 1:** Sociodemographic characteristics.

	DBE N = 192(%)	NDBE N = 213 (%)	Reflux N = 111 (%)	*P*
Male gender	161 (83.9)	149 (69.6)	40 (36.0)	< 0.01
Age in years *mean (SD)*	70.9 (9.1)	63.3 (8.9)	60.4 (16.8)	< 0.01
Marital status				< 0.01
No relationship	25 (13.0)	17 (8.0)	31 (27.9)	
Married/living together	145 (75.5)	178 (83.6)	71 (64.0)	
Divorced	1 (0.5)	9 (4.2)	2 (1.8)	
Widow/ widower	21 (10.9)	9 (4.2)	7 (6.3)	
Employment status				< 0.01
Employed	48 (25.0)	90 (42.3)	51 (45.9)	
Unemployed	19 (9.9)	23 (10.8)	19 (17.1)	
Retired	125 (65.1)	100 (46.9)	41 (36.9)	
Total comorbidity *mean (SD)*	2.7 (1.9)	2.4 (2.0)	1.8 (1.6)	< 0.01
Positive history of cancer	55 (30.4)	26 (12.2)	13 (11.7)	< 0.001
Positive family history with cancer	36 (18.8)	69 (32.4)	39 (36.1)	0.002
Months after ET		n.a	n.a	na
0–5	13 (6.8)			
06-Nov	53 (27.6)			
Dec-35	31 (16.1)			
> 36	95 (49.5)			
Worst pathology			n.a	na
NDBE	–	213 (100)		
LGD	54 (28.1)			
HGD	46 (24.0)			
EAC	83 (43.2)			
High risk EAC	9 (4.7)			
Length BE	n.a		n.a	na
1-3 cm		103 (48.4)		
4-9 cm		82 (38.5)		
> 10 cm		28 (13.1)		
Dyspepsia*				< 0.001
None	152 (79.2)	170 (79.8)	41 (36.9)	
Mild	32 (16.7)	40 (18.8)	36 (32.4)	
Severe	8 (4.2)	3 (1.4)	34 (30.6)	
Regurgitation*				< 0.001
None	141 (73.4)	146 (68.5)	46 (41.4)	
Mild	44 (22.9)	53 (24.9)	37 (33.3)	
Severe	7 (3.6)	14 (6.6)	28 (25.2)	
Heartburn*				< 0.001
None	147 (76.3)	168 (78.9)	27 (24.3)	
Mild	34 (17.7)	42 (19.7)	53 (47.7)	
Severe	11 (5.9)	3 (1.4)	31 (27.9)	
Total score RDQ*				< 0.001
None	149 (77.6)	163(76.5)	25 (22.5)	
Mild	40 (20.8)	47 (22.1)	59 (53.2)	
Severe	3 (1.6)	3 (1.4)	27 (24.3)	

Results are described with N (%) DBE: dysplastic Barrett Esophagus, NDBE: non- dysplastic Barrett Esophagus, BE: Barrett esophagus, ET: Endoscopic treatment, LGD: Low grade dysplasia, HGD: high grade dysplasia, EAC: esophageal adenocarcinoma. RDQ: Reflux Disease Questionnaire *A score of none represent a score of 0 on the RDQ, mild symptoms a score of 1–2, and severe 3–5.

The reflux group contained of 111 refractory reflux patients. The mean age of the reflux group was 60.2 years (SD = 16.8 years) and 36% were male. This group statistical significantly differed from the BE group on all sociodemographic characteristics. The reflux patients were predominately female and statistical significant younger than in comparison to the BE group. With 1.8 (SD 1.6) comorbidities per participant, reflux patients had fewer comorbidities in contrast to the 2.6 (SD 1.9) in de BE group.

**Figure 1:**
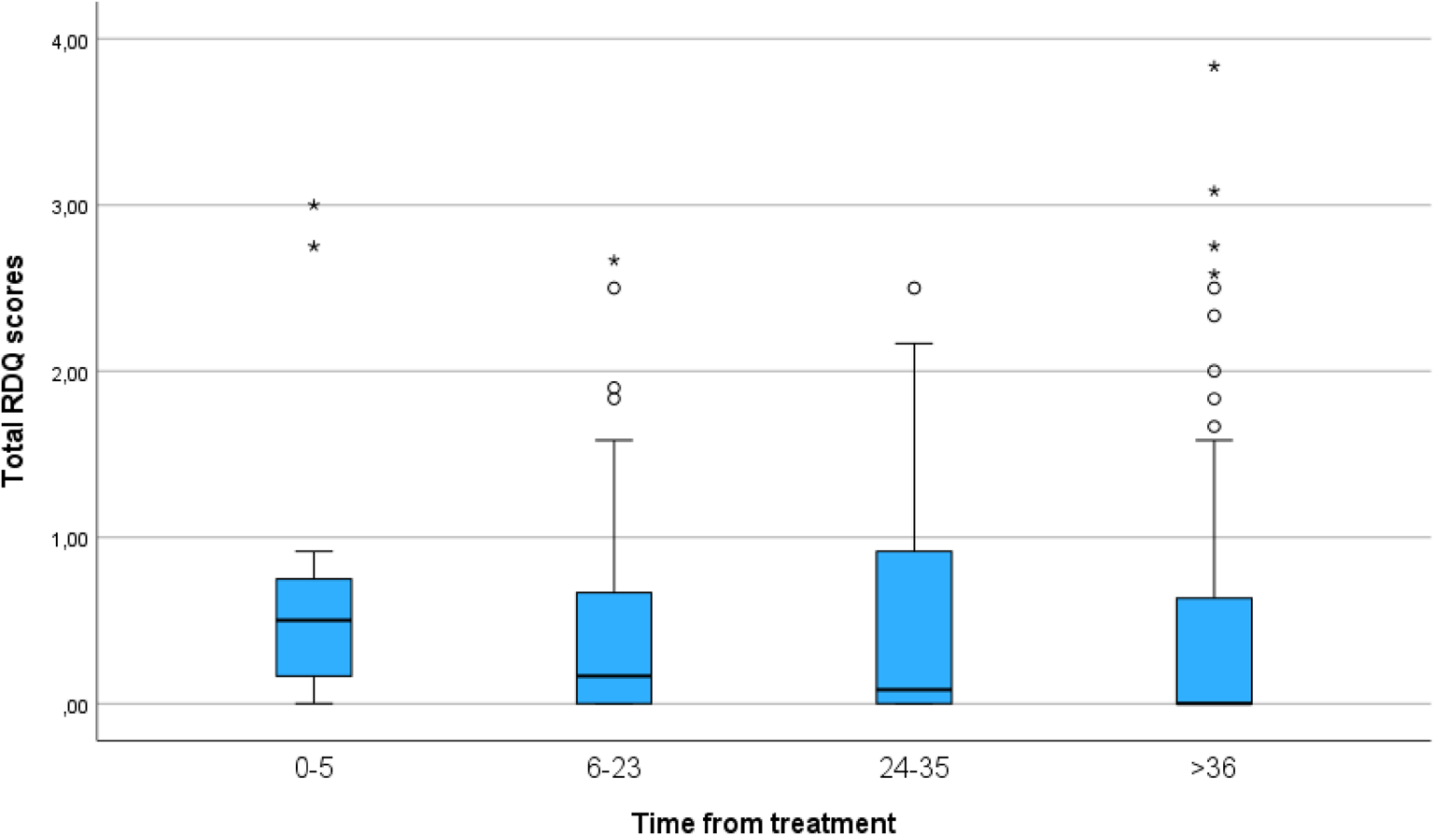
Reflux symptoms versus time after the last endoscopic treatment, measured with the Reflux Disease Questionnaire (RDQ). A score of 0 represent nil symptoms, a score of 1–2 mild symptoms, and 3–5 severe symptoms of reflux.

When questioned whether DBE patients experienced reflux symptoms in the last seven days, 77.6% of the patients reported that they had experienced none (Fig. 1). There were significant more GI symptoms (e.g. heartburn, dyspepsia and regurgitation) in the reflux group in comparison with the two BE groups (t(514) -15.68 = *p* =  < 0.001). In which 24.3% of the reflux patients versus 1.5% in the BE patients were experiencing severe reflux symptoms. Patients currently under ET tended to have more regurgitation and dyspepsia symptoms compared to previously treated patients, however this difference was not statistically significant.

### Cancer worry

Table 2 shows that both BE groups scored low on mean cancer worry (i.e. NDBE 9.13 and DBE 9.19). Comparison of mean cancer worry scores between the BE groups showed no statistically significant differences (X^2^ (2), N = 400) = 0.880, *p* = 0.644). The reflux patients scored statistically significant worse on cancer worry in comparison to BE patients. Specifically, comparison of the level of high cancer worry between groups showed 56.6% of reflux patients versus 40.6% of the DBE and 36.2% of NDBE stated high cancer worry (X^2^ (2 N = 495) = 21.8, *p* =  < 0.001).

**Table 2:** Cancer worry measured with the Cancer Worry Scale.

	DBE	NDBE	Reflux	*P*
Total cancer worry mean (SD)	9.19 (2.9)	9.13 (3.0)	10.28 (3.5)	0.004
No cancer worry	42 (22.5) _a_	50 (23.5)_a_	18 (17.0)_a_	< 0.001
Low cancer worry	69 (36.9)_a ,b_	86 (40.4)_a_	28 (26.4)_b_	
High cancer worry	76 (40.6)_a_	77 (36.2)_a_	60 (56.6)_b_	

Results are described with N (%). A p-value < 0.05 was considered statistically significant.

Each subscript letter denotes a subset of patient categories whose column proportions do not differ significantly from each other at the 0.05 level. DBE: dysplastic Barrett Esophagus, NDBE: non- dysplastic Barrett Esophagus.

**Figure 2:**
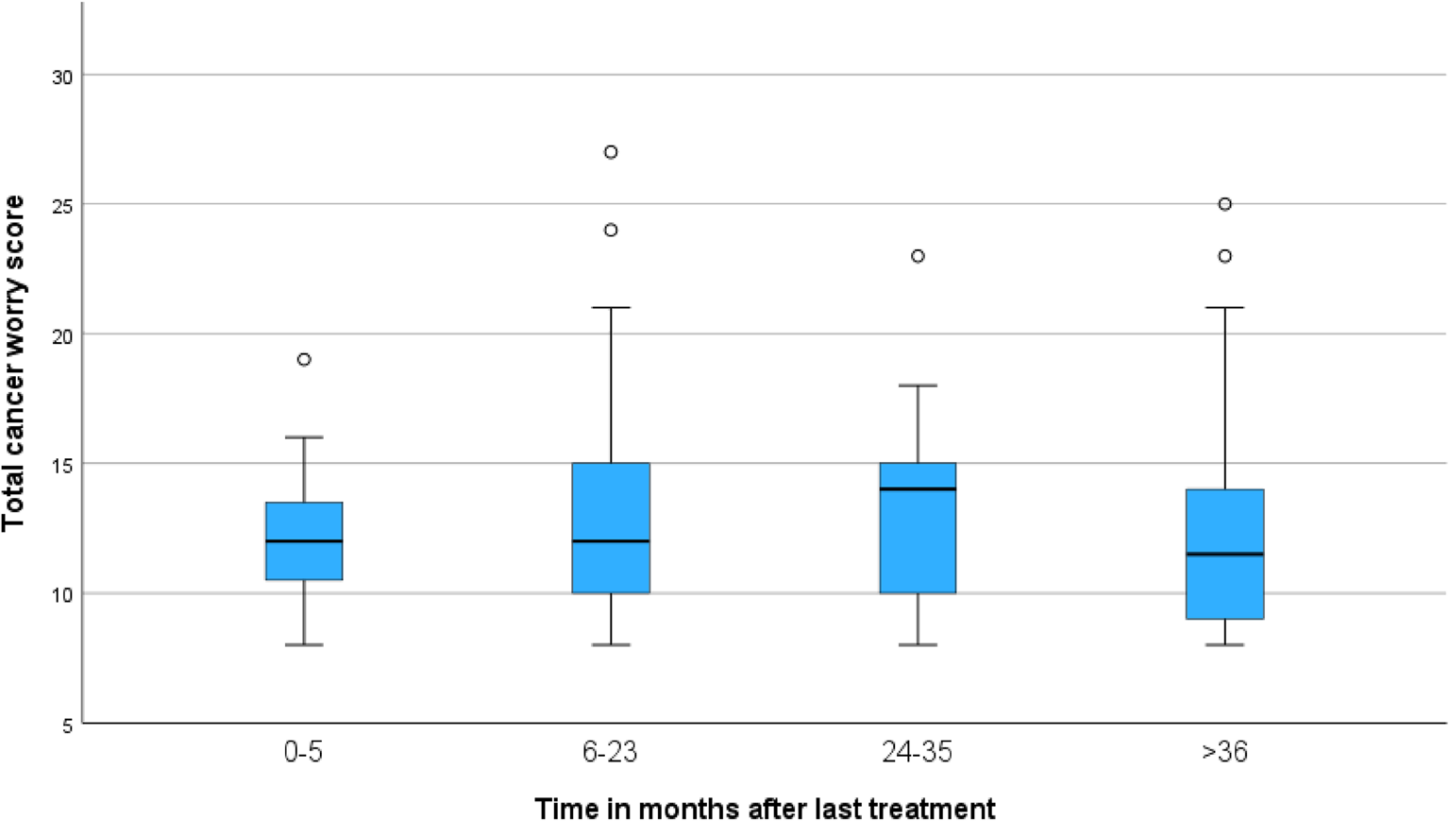
Comparison of cancer worry versus time after the last endoscopic treatment, measured with the cancer worry scale. *S*cores of cancer worry did not change over time after the last endoscopic treatment (F (3,183) = ,598 *p* = 0.617).

Of the patients endoscopically treated for EAC, only 33% reported they had cancer treatment in their medical history and 44.4% of the patients with high-risk EAC (lymfovascular invasion or > sm1) stated they were treated for cancer in the past. As shown in Fig. 2, scores of cancer worry did not correlate with time after the last endoscopic treatment (r = −.048; *p* = 0.522 N 180).

### Associated factors

In the DBE group, a younger age had a low negative correlation with higher scores on cancer worry (r = − .190; *p* = 0.009 N 187). A DBE patient with a family or friend with a positive history of EAC scored higher on cancer worry (r = 0.192; *p* = 0.008 N 187). Likewise, having a medical history of cancer had a small negative correlation with more cancer worry in this group (r = 0.153; *p* = 0.037 N 187). The grade of histology and time from ET was not associated with higher scores on cancer worry. There was a moderate positive correlation found between the reflux symptoms and cancer worry in the NDBE group (r = 0.326; *p* =  < 0.000 N 213) and a low correlation in the reflux group (r = 0.233; *p* = 0.019 N 111). This correlation was not found in de DBE group (r = 0.136; *p* = 0.063 N 187). There was no association found between the NDBE length and cancer worry (r = 0.460, *p* = 0.051 N 213).

## Discussion

Although the chance of cancer progression in a Barrett’s esophagus is very low, worry for cancer is known as an important factor influencing HRQoL and negative perceptions of the diagnosis BE^14^. In the present study, we determined the proportion of BE patients with high levels of worry for cancer and aimed to compare outcomes between patients endoscopically treated for BE neoplasia, non-dysplastic BE patients and patients with reflux symptoms. We hypothesized that the minority of BE patients would experience high cancer worry which would be associated with physical symptoms and not related to factors that would actually increase the risk of cancer such as Barrett’s length or histology outcomes.

Overall, BE patients reported a low mean score on cancer worry, however still 40.6% of the DBE patients and 36.2% of the NDBE patients stated high cancer worry. In line with our results, a study from the UK showed no differences between cancer worry in a DBE group and NDBE group^11^. However, overall CWS scores of the BE groups in the UK study were higher (more cancer worry) than in the present study, specifically a mean of 12.8 in the UK patients versus 9.2 in the present study was found. The reason for this difference is not clear, but it might be related to the differences in care pathways, lower levels of education in the UK group. Although baseline characteristics of the two studies seemed to correspond, education level and ethnicity could be involved, but were not reported.

A possible explanation for the fact that the reflux patients in the present study were experiencing higher levels of worry for cancer than BE patients, is the fact that the data was used of patients with reflux symptoms refractory for PPI prior to their first upper GI endoscopy. Consequently, it is possible that these patients were more concerned about cancer because they missed the reassurance of an upper GI endoscopy. Previous studies have shown that BE patients felt a sense of control after undergoing upper GI endoscopy, which may have had a positive effect on cancer worries^22,23^. The presence of high cancer worry in a group of patients with refractory reflux symptoms supports the hypothesis that experiencing reflux symptoms is related to worry for cancer. Experiencing reflux symptoms was moderately correlated with more cancer worry in the NDBE group and reflux group, this linear correlation was not found in the DBE group. Theoretical models of fear of cancer recurrence propose that somatic symptoms can trigger fear^24,25^. Studies have consistently found that higher prevalence of post cancer symptoms is associated with greater fear of cancer recurrence^26,27^. Furthermore, it has been demonstrated that experiencing symptoms of dysphagia, dyspepsia or heartburn in BE patients is associated with more fear of cancer^11,14^.

Because reflux symptoms in BE patients appear to be an important factor in relation to worry for cancer, we further explored the prevalence and intensity of reflux symptoms. In the majority of DBE patients reflux symptoms were comparable with those with NDBE, and represent a good symptom control. Consistent with the literature^28,29^, this study found that refractory reflux patients reported statistical significantly more reflux symptoms than BE patients. A possible explanation for these results may be the lack of esophageal sensitivity in BE patients instigated by significantly reduced esophageal acid sensitivity and an impaired ability to recognize acid reflux^30^. A second explanation could be the inadequate symptom control by the PPI prescribed. Although all reflux patients used a standard dose of PPI therapy for at least 3 times a week during a minimum of three months. It could be expected that the BE population had better PPI doses regulations then the reflux population who had been referred with refractory reflux symptoms. In addition to the impact on cancer worry, GERD has been associated with functional deficiencies, such as sleep difficulties, reduced ability to consume food, impaired sex life, thus affecting quality of life and increasing the risk for a comorbid mental disorder^31,32^. A previous study showed patients with BE have better disease-specific HRQoL when compared to patients with GERD. This difference was partially attributable to lower symptom severity amongst BE patients^33^. Appropriately adjusted medical treatment is essential for reducing GERD related symptoms.

To the best of our knowledge, this was the first study exploring factors associated with worry for cancer in BE patients. In addition to the association between reflux symptoms and worry for cancer, there was an association found between a younger age and high cancer worry in BE patients treated for (early) neoplasia. Previous research in cancer survivors have found that a younger age was a prominent factor associated with higher fear of cancer^26,34^. The underlying causes have not been determined, but the perception that cancer threatens the achievement of certain important life projects (e.g., career and marriage or having children) may play a role.

In DBE patients with a family or friend with a positive history of EAC, a higher cancer worry was found. This was in contrast with a review on fear of cancer recurrence in adult cancer survivors, which concluded that a family history of cancer was not associated with an increased fear of cancer^26^. Previous research in BE patients found patients with a friend or family member with cancer, were more likely to overestimate their risk for EAC^7^. Furthermore, there is some evidence that family caregivers report higher levels of fear of cancer than survivors^35^. As a physician, it is important to be aware of increased cancer worry if cancer is present in a family or friend or in their own medical history.

There was no correlation found between the degree of histology and the level of worry for cancer. Surprisingly, only 33% of the patients endoscopically treated for EAC, reported they had cancer treatment in their medical history. Of the patients with high-risk EAC (lymfovascular invasion or > sm1), this was 44.4%. A possible explanation for this might be that patients were associating a cancer treatment—or even the word cancer— with death and trepidation^2^. Endoscopic resection is the first-choice therapy for T1a EAC and is minimally invasive compared with surgical treatment. And therefore, this minimal invasive treatment may not be perceived as a cancer treatment. An important contributing factor is the possible lack of patient knowledge, specifically about histology outcomes. A previous qualitative study reported poor disease-specific knowledge in BE patients^36^. Thus, patient education needs to be comprehensive and easily understood.

Furthermore, there were no correlations found between the time after ET and the level of cancer worry. This in contrast to the studies of Shaheen and Rosmolen et al.^9,12^, who found that post- ET cancer worry declined over time. There are several explanations for this difference. First, the cross-sectional design in the present study, could not demonstrate a change in scores of an individual patient. All we could demonstrate is that the mean scores of patients directly after ET and of patient’s years afterwards do not vary. Additionally, the results of the two studies may not be comparable because different measurement instruments were used.

Three notable limitations affected this study. The first limitation was the cross-sectional design of this study, as a result change over time within an individual patient could not be detected. Further research with a longitudinal prospective design would determinate the true development of cancer worry over time. Second, the study was partly conducted during the Covid pandemic, which may have contributed to the patient's responses, although implementation of lockdown was not there during the data collection phase. However, a previously conducted sensitivity analysis showed no difference on primary and secondary outcomes before, during and after the COVID period. Third, this is an exploratory study, for this reason our findings are in need of replication before they can be accepted with confidence. Finally, no questionnaire was used on psychological distress, which is known to be an important general factor influencing worry for cancer.

The findings of this study have a number implications for daily practice. First, BE patients experiencing reflux related symptoms should receive adequate treatment. Furthermore, BE patients should receive adequate information on the diagnosis BE and the actual minor cancer risk. If high levels of cancer worries are persistent, cognitive behavioral therapy can be considered. Psychological interventions with cognitive behavioral therapy for fear of cancer recurrence revealed a small but robust effect at post intervention, which was largely maintained at follow-up^37^.

In the present study, a significant group of BE patients reported high cancer worry which was associated with reflux symptoms in NDBE patients and a younger age, and a (family) history of the diagnosis esophageal carcinoma in BE patients treated for (early) neoplasia. Physicians should communicate about the actual cancer risk unambiguously, which leads to greater patient understanding and may therefore positively affects health outcomes.

## Data Availability

The datasets used and/or analyzed during the current study is available from the corresponding author on reasonable request.
